# The ensemble of gene regulatory networks at mutation–selection balance

**DOI:** 10.1098/rsif.2022.0075

**Published:** 2023-01-04

**Authors:** Chia-Hung Yang, Samuel V. Scarpino

**Affiliations:** ^1^ Network Science Institute, Northeastern University, Boston, MA, USA; ^2^ Institute for Experiential AI, Northeastern University, Boston, MA, USA; ^3^ Department of Health Sciences, Northeastern University, Boston, MA, USA; ^4^ Khoury College of Computer Sciences, Northeastern University, Boston, MA, USA; ^5^ Roux Institute, Northeastern University, Boston, MA, USA; ^6^ Santa Fe Institute, Santa Fe, NM, USA; ^7^ Vermont Complex Systems Center, University of Vermont, Burlington, VT, USA

**Keywords:** gene regulatory networks, quasi-species theory, mutation–selection balance, neutral network

## Abstract

The evolution of diverse phenotypes both involves and is constrained by molecular interaction networks. When these networks influence patterns of expression, we refer to them as gene regulatory networks (GRNs). Here, we develop a model of GRN evolution analogous to work from quasi-species theory, which is itself essentially the mutation–selection balance model from classical population genetics extended to multiple loci. With this GRN model, we prove that—across a broad spectrum of selection pressures—the dynamics converge to a stationary distribution over GRNs. Next, we show from first principles how the frequency of GRNs at equilibrium is related to the topology of the genotype network, in particular, via a specific network centrality measure termed the eigenvector centrality. Finally, we determine the structural characteristics of GRNs that are favoured in response to a range of selective environments and mutational constraints. Our work connects GRN evolution to quasi-species theory—and thus to classical populations genetics—providing a mechanistic explanation for the observed distribution of GRNs evolving in response to various evolutionary forces, and shows how complex fitness landscapes can emerge from simple evolutionary rules.

## 1. Introduction

Molecular networks influence both macro- and micro-evolutionary processes [1–5]. But, how might they themselves evolve? A recent comparative study of regulatory networks found that their structures often exist at the edge of critically, straddling the border of chaotic and ordered states [6]. The idea that biological regulatory networks should exhibit the kind of dynamic stability associated with near-critical networks has been theorized as adaptive, both from the perspective of maintaining functionality under mutational perturbation, i.e. their robustness [7,8], and their ability to effectively process information [9]. However, there is also substantial empirical and theoretical evidence for the importance of evolutionary change in these networks, i.e. their so-called evolvability [8,10]. Indeed, a trade-off between robustness and evolvability is hypothesized as an explanation for the common ‘small-world’ property—also a feature of near-critical networks—seen in biological networks [11]. Nevertheless, foundational work on self-organized criticality and 1/*f* noise demonstrated that dynamical systems embedded in a spatial dimension, e.g. biological regulatory networks, might naturally evolve to near-critical states [12,13].

Over the past two decades, several models of gene regulatory network (GRN) evolution have been proposed [14–19], which have influenced our understanding of diverse phenomena including canalization [15,20], allopatric speciation [17,18,21], expression noise [22] and the structural properties of GRNs themselves [16,23,24]. Empirical studies of transcription factors [25,26], mRNA profiles [27] and comparative genomics [28] suggest that gene duplication/loss and modularity both play an essential role in generating the observed patterns of gene regulation [29–31]. Building from these studies, Force *et al.* [30] showed computationally that the subfunctionalization of ancestral genes following duplication events can lead to modular GRNs. Similarly, Espinosa-Soto & Wagner [24] demonstrated that sequential adaptation via newly specialized gene activity patterns can increase the modularity of GRNs.

More recently, studies integrating empirical data with computational models have hypothesized how an expanded set of evolutionary forces may shape the structure of GRNs [32–35]. The results of this work are supported by several mathematical models of GRN evolution introduced to study regulatory structures influenced by duplication events [36], selection on functional dynamics [37], horizontal gene transfer [38], correlated mutations [39] and non-genetic inheritance [40]. Conversely, GRNs are hypothesized to evolve largely as a by-product of the progression towards some optimal state, through a combination of negative-feedback regulation [41], the rate of molecular evolution [42], trade-offs between robustness and evolvability [6] and self-organization of functional activity [43].

When genotypes are expanded to more complicated sets of genes, the long-term stationary solution for a population genetic model describing their evolution can be studied with quasi-species theory [44,45]. Using this approach, prior work has derived exact solutions for the steady-state distribution of higher dimensional genotypes and criteria for global convergence [46–48]. However, results obtained in these studies relied on an assumption of mutational accessibility among genotypes with non-zero fitness, i.e. irreducible and primitive transition matrices, and often on particular functional forms for selection [49–53]. Demonstrating analytically that mutations are accessible and that the steady-state distribution of GRNs can be studied under arbitrary forms of selections will advance both our theoretical understanding of how these structures evolve and capacity to evaluate theory with empirical data.

Here, we derive analytical conclusions for models of GRN evolution using an approach similar to those taken in population genetics and quasi-species theory [45]. However, instead of assuming mutational accessibility, we prove its existence mathematically. Specifically, we study the dynamics of GRN evolution in an infinitely large population with non-overlapping generations at mutation/selection balance. Using this model, we mechanistically recover empirical observations such as GRN modularity and prove that the dynamics always converge to a stationary distribution over GRNs. Then, assuming binary viability, identical ability to reproduce and rare mutation, we analytically show that the frequency of GRNs at mutation–selection balance is proportional to each GRN’s eigenvector centrality in a sub-graph of the genotype network [54–57]. Finally, we determine which structural motifs associated with GRNs are favoured in response to a wide variety of selective regimes and regulatory constraints. We discuss how analysing GRN evolution using a network-science approach can provide a mechanistic explanation for the way evolution shapes and is constrained by higher-order interactions [58].

## 2. Models

### 2.1. Quasi-species model with selection, reproduction and mutation

**Figure 1.**
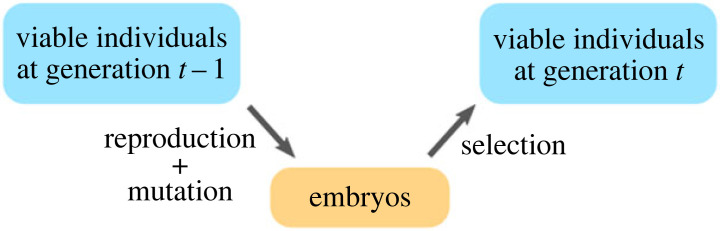
Illustrative cartoon of different stages in the proposed quasi-species model.

We begin with a quasi-species model that incorporates selection, asexual reproduction and mutation such that the viable individuals in the current generation reproduce and generate their offspring, which experience mutation and undergo selection to form the next generation (see figure 1 for a simple sketch). This phenomenological modelling scheme is quite common for studying both deterministic and stochastic Markovian dynamics [44,46–48,59]. Yet, as we show shortly, basic probability theory allows us to construct a model from the bottom up and provides a probabilistic interpretation of the various parameters. Additionally, we impose a few key assumptions, including: (i) an infinitely large population size, (ii) non-overlapping generations, (iii) asexual reproduction (i.e. no random assortment nor recombination), (iv) a fixed selective environment, and (v) that any single-locus mutation has a non-zero chance to occur per generation.

More formally, suppose that *I*_*t*_ represents an individual randomly sampled from the population at generation *t*. Let *g*(*I*_*t*_) be its genotype and $\Psi (I_{t})$ the random variable of whether *I*_*t*_ is viable. Here $\Psi (I_{t})=1$ represents the event that *I*_*t*_ survives, and $\Psi (I_{t})=0$ otherwise. We further denote by *I*_*t*−1_ → *I*_*t*_ the event that the randomly sampled individual at generation *t* − 1, namely *I*_*t*−1_, reproduced and generated the randomly sampled individual at generation *t*, namely *I*_*t*_. We use G to represent the set of all plausible genotypes.

For any genotype g∈G, we are interested in its prevalence in the population at a given generation *t* *after* selection. In other words, we would like to know the probability that we observe a randomly sampled individual at generation *t* with genotype *g*, given that the sampled individual is viable. Applying Bayes’ theorem, this focal conditional probability becomes

2.1 $$\begin{array}{rl} P[g(I_{t})=g|\Psi (I_{t})=1] & =\frac{P[\Psi (I_{t})=1|g(I_{t})=g]P[g(I_{t})=g]}{P[\Psi (I_{t})=1]}\\ & =\frac{\nu_{g}}{\nu_{t}}P[g(I_{t})=g]. \end{array}$$

For simplicity, we adopt the abbreviation $\nu_{g}=P[\Psi (I_{t})=$ $1|g(I_{t})=g]$ and $\nu_{t}=P[\Psi (I_{t})=1]$, which are equivalently the survival probability or the *viability* of genotype *g*, and the average viability at generation *t*.

What we have left in equation (2.1) is the probability that a randomly sampled individual has genotype *g* *before* selection. The derivation of $P[g(I_{t})=g]$ relies on two observations: first, the genotype of individual *I*_*t*_ depends on the unique genotype of its parent and any subsequent mutation; second, this parent individual must be viable. The event *g*(*I*_*t*_) = *g* is hence partitioned^1^ by the joint events ${\left\{g(I_{t})=g,g(I_{t-1})=g^{\prime }|I_{t-1}\to I_{t},\Psi (I_{t-1})=1\right\}}_{g^{\prime }\in G}$. So we have

2.2 $$\begin{array}{rl} P[g(I_{t})=g] & =\sum_{g^{\prime }\in G}P[g(I_{t})=g,g(I_{t-1})=g^{\prime }|I_{t-1}\to I_{t},\Psi (I_{t-1})=1]\\ & =\sum_{g^{\prime }\in G}P[g(I_{t})=g|g(I_{t-1})=g^{\prime },I_{t-1}\to I_{t},\Psi (I_{t-1})=1]\\ & \;\times P[g(I_{t-1})=g^{\prime }|I_{t-1}\to I_{t},\Psi (I_{t-1})=1]\\ & =\sum_{g^{\prime }\in G}\mu_{g^{\prime }g}P[g(I_{t-1})=g^{\prime }|I_{t-1}\to I_{t},\Psi (I_{t-1})=1]. \end{array}$$

We abbreviate $\mu_{g^{\prime }g}=P[g(I_{t})=g|g(I_{t-1})=g^{\prime },I_{t-1}\to I_{t},$ $\Psi (I_{t-1})=1]$, which shows the *mutational probability* from genotype *g*′ to genotype *g*.

Finally, $P[g(I_{t-1})=g^{\prime }|I_{t-1}\to I_{t},\Psi (I_{t-1})=1]$ in equation (2.2) is the probability that the parent of a randomly sampled individual at generation *t* has genotype *g*′. Applying Bayes’ theorem once more, this probability becomes

2.3 $$\begin{array}{rl} P[g(I_{t-1})=g^{\prime }|I_{t-1}\to I_{t},\Psi (I_{t-1})=1] & =\frac{P[I_{t-1}\to I_{t}|g(I_{t-1})=g^{\prime },\Psi (I_{t-1})=1]P[g(I_{t-1})=g^{\prime }|\Psi (I_{t-1})=1]}{P[I_{t-1}\to I_{t}|\Psi (I_{t-1})=1]}\\ & =\frac{\rho_{g^{\prime }}}{\rho_{t-1}}P[g(I_{t-1})=g^{\prime }|\Psi (I_{t-1})=1], \end{array}$$

where $\rho_{g^{\prime }}=P[I_{t-1}\to I_{t}|g(I_{t-1})=g^{\prime },\Psi (I_{t-1})=1]$ is the *reproductivity* of genotype *g*′, and $\rho_{t-1}=P[I_{t-1}\to I_{t}|\Psi (I_{t-1})=1]$ is the average reproductivity at generation *t* − 1. Note that, instead of defining reproductivity as the number of offspring an individual has, the probabilistic formulation describes reproductivity as—when sampling from the infinitely sized next generation—how likely is one to observe an offspring of the focal genotype.

More importantly, we see that equation (2.3) leads us back to the conditional probability that, at generation *t* − 1, a randomly sampled individual has genotype *g*′ given that it is viable. Combining (2.1) to (2.3), we obtain the master equation for a quasi-species model that integrates selection, reproduction and mutation,

2.4 $$\begin{array}{rl} P[g(I_{t}) & =g|\Psi (I_{t})=1]\\ & =\frac{1}{\nu_{t}\rho_{t-1}}\sum_{g^{\prime }\in G}\rho_{g^{\prime }}\mu_{g^{\prime }g}\nu_{g}P[g(I_{t-1})=g^{\prime }|\Psi (I_{t-1})=1]. \end{array}$$

### 2.2. Pathway framework of gene regulatory networks: representing genotypes by expression behaviour

In the existing literature on quasi-species theory, the model parameters are usually arbitrarily tunable or follow an assumed distribution for simplicity. Hypothetically, these parameters depend on the resultant phenotypes of the genotypes, and any genotype–phenotype mapping reflects constraints and provides information on the model parameters. In previous work, we proposed a modelling approach, termed the *pathway framework*, to describe how the structure of GRNs varies due to genetic changes and how they respond to a given selective pressure [18,19] (which we summarize below; see its formal mathematical formulation in electronic supplementary material, appendix A). In the current work, we apply the pathway framework of GRNs as a genotype–phenotype mapping for parametrizing a quasi-species model (2.4).

**Figure 2.**
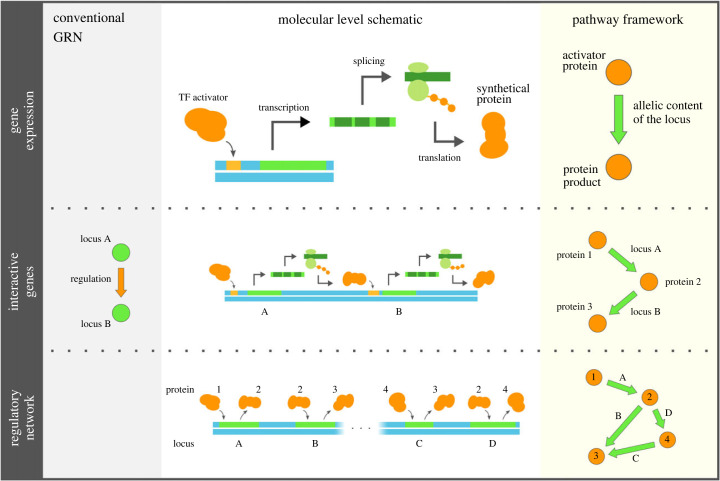
The pathway framework interprets a GRN as an abstraction of the expression behaviour of the genotype. In this framework, a GRN consists of edges indicating the input–output pair of a gene’s expression, from which transcriptional regulation between genes can be recovered, and it is arguably a more compact representation than the conventional notion of GRNs.

The pathway framework conceptualizes alleles of genes as ‘black boxes’ that encapsulate their expression behaviour. Regulation between two genes naturally arises when one gene’s protein product is involved in the activation of the other’s expression (see figure 2). The pathway framework, therefore, represents the genotype as the input–output relation of each gene’s expression behaviour, and the corresponding GRN can be constructed by connecting genes based on regulation. The collective state of each network is the resulting genotype. As a consequence, these input–output relations of gene expression serve as the ‘inherited’ reactions, where the external environmental stimuli trigger an expression cascade that activates proteins. We consider the final state of the network following such a cascade the GRN’s phenotype.

We note that the pathway framework is not without a handful of assumptions and thus may be limited to specific GRNs. Nevertheless, in the pathway framework GRNs play the role of an informative genotype–phenotype mapping, which evokes some mechanistically interpretable parametrization for models in quasi-species theory. In later sections we show how, despite this somewhat naive and specific genotype–phenotype mapping, we obtain a fairly broad demonstration of the key assumption in quasi-species theory—e.g. mutational accessibility—when proving the global convergence to a stationary solution (see §4).

In this work, we focus on a minimal pathway framework of GRNs which relies on a few additional assumptions. First, we assume there is a constant collection of proteins Ω that can *possibly* appear in the organisms, and the state of a protein is binary, which indicates whether the protein is present or absent in an organism. Second, assuming that any gene’s expression is activated by a single protein and produces a single protein product, the allele of the gene becomes the ordered pair of protein activator/product. If the protein activator is in the present state, the allele of the gene turns the state of the protein product to present. Third, there is a fixed collection of genes Γ in the organisms, and the allele of each gene can be any pair of activator/product in the constant collection of proteins. Fourth, the external environmental stimuli, if any, specify some activator proteins in the constant collection Ω and sets their state to present.

Under these assumptions, a GRN can be transformed from its conventional notion, where nodes in the network represent genes and the edges show regulation among them, into a more compact format such that the nodes are exactly the constant collection of proteins, and the directed edges describe the expression behaviour of alleles of genes (see figure 2). Hereafter, we use the term GRN to describe those networks represented in this more compact format under the pathway framework. However, we note that the two constructions are merely different representations of the expression behaviour of the same underlying genotype. While the set of all possible genotypes is denoted by G in §2.1, because the pathway framework assumes that each gene is represented by its expression behaviour and the GRN is constructed through chains of expression, we also use the notation G for their corresponding GRNs, and we write g∈G to refer to a possible genotype/GRN. Given the constant collection of proteins Ω and genes Γ, the set of all possible GRNs is determined such that each possible GRN is a network among Ω with |Γ| directed edges, each of which is labelled by a gene in Γ and points from any protein activator to any protein product in Ω.

The pathway framework provides an approach to model various evolutionary forces, such as random mutation and natural selection, through graphical operations and structural characteristics on the GRNs. Mutation at a gene changes its allele stochastically, which is essentially a random process over all possible pairs of protein activators/products in the constant collection Ω excluding the original allele. In the corresponding GRN, mutating a gene is equivalent to rewiring the directed edge labelled by the focal gene. On the other hand, selection is usually characterized as some phenotypic response evaluated against an environment. In this case, because a phenotype is developed through a cascade of internal protein expression starting from the external stimuli, the binary state of a protein in the resulting phenotype corresponds to its reachability from the stimulated proteins in the GRN. The viability and the reproductivity of a genotype can, therefore, be modelled as functions of node reachability in the GRN. For example, in the case study in §3.2, we will consider a simple scenario where the mutation at each gene is independent and the outcome is uniform among all possible alleles, and that the viability is 1 if some phenotypic constraint is satisfied or 0 otherwise. We explore more complex scenarios in later sections.

### 2.3. Genotype networks: a space of mutational relationship between gene regulatory networks

**Figure 3.**
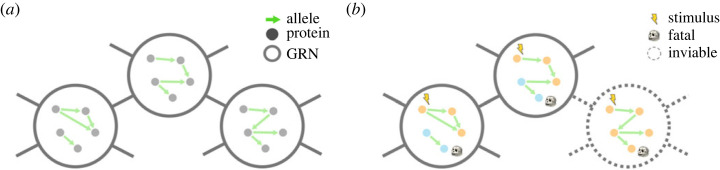
(*a*) Genotype network of GRNs, where, under the pathway framework, two mega-nodes (GRNs) are connected if and only if thy differ by one edge rewiring. (*b*) Neutral network of GRNs, where inviable mega-nodes are removed from the genotype network. In this illustrative example inviability is modelled as a regulatory pathway from the stimulus to the protein product with a fatal effect.

Previous work has developed the concept of the genotype network, which captures how various genotypes transition from one to another through mutations (not necessarily just point mutations) and/or recombination [54,60,61]. Here, we adopt the genotype network to describe the mutational connection between GRNs. The *genotype network* of GRNs is a undirected network of networks, where every possible GRN becomes a mega-node, and two mega-nodes are connected if the two corresponding GRNs differ only by an allele at a single locus. In other words, an edge between two mega-nodes in the genotype network represents a single-locus mutation between GRNs (figure 3). Instead of concentrating on all possible GRNs, we often focus on the mutational relationship between a subset of GRNs. One of the most common phenotypic constraints is to only consider GRNs with equal viability and reproductivity, where the induced sub-graph of the genotype network is known as the *neutral network* [56,61], which captures mutational transitions between GRNs that are selectively neutral in a single generation.

We emphasize two important properties of a genotype network of GRNs and its induced sub-graphs under the pathway framework. First, because the underlying collections of proteins and genes are fixed, and a mutation at any gene can lead to a mutant allele that points from any protein activator to any protein product, each GRN has the same number of mutational neighbours. As a result, the genotype network of GRNs is in fact a regular graph. Second, for any phenotypic constraint, we show that the resulting induced sub-graph of the genotype network is connected. In other words, there always exists a sequence of single-locus mutations between two GRNs such that the involved GRNs all satisfy the arbitrarily given constraint on their phenotype. The guaranteed connectedness also applies to the neutral network of GRNs, where the phenotypic constraint corresponds to protein states leading to the same viability and reproductivity.

We leave to electronic supplementary material, appendix C the detailed proof for the connectedness of a sub-graph of the genotype network induced from arbitrary phenotypic constraint, but provide a brief outline here. The proof is based on a few observations of the pathway framework of GRNs. Under the assumption of binary protein states, there naturally exist some protein activator/product pairs that are ‘redundant’ in terms of the resulting phenotype. Such redundancy manifests when the product is simply the activator itself, or when multiple genes share the same activator/product pair. Furthermore, given a phenotypic constraint, one can come up with a family of ‘naive’ GRNs that satisfy the constraint. Specifically, such a ‘naive’ GRN is constructed by (i) for each required-present protein, assigning it as the product of a gene with an external stimulus as the activator and (ii) assigning the rest of genes with redundant activator/product pairs. Our proof in electronic supplementary material, appendix C systematically finds a mutational trajectory between two GRNs *g*_*s*_ and *g*_*t*_ satisfying the phenotypic constraint. This trajectory consists of three segments—between *g*_*s*_ and a naive GRN *g*′, between *g*′ and another naive GRN *g*″, and finally between *g*″ and *g*_*t*_—and all GRNs traversed by the mutational trajectory also satisfy the given phenotypic constraint.

## 3. Analyses

### 3.1. Convergence to a stationary distribution of gene regulatory networks

Our goal now is to prove the convergence of the quasi-species model (2.4) under the pathway framework and derive the stationary distribution over possible GRNs at mutation/selection balance. We begin by focusing on groups of GRNs whose probability to be observed is relatively straightforward to model. First, for any GRN *g* with a zero viability, i.e. $\nu_{g}=P[\Psi (I_{t})=1|g(I_{t})=g]=0$, the probability to observe *g* from a randomly sampled individual that has survived selection is also zero. Formally speaking, denoting those GRNs with a non-zero viability by $G_{v}=\{g\in G|\nu_{g}>0\}$, we have $P[g(I_{t})=g|\Psi (I_{t})=1]=0$ for each $g\in G\setminus G_{v}$ and at any time *t*. Second, we denote the GRNs with a zero reproductivity by $G_{s}=\{g\in G|\rho_{g}=0\}$. Since GRNs $G_{s}$ do not contribute offspring, their probability to be observed solely depends on the other GRNs $G\setminus G_{s}$. In particular, for each $g\in G_{s}$ and at any time *t*, we have $P[g(I_{t})=g|\Psi (I_{t})=1]=(1/\nu_{t}\rho_{t-1})$ $\sum_{g^{\prime }\in G\setminus G_{s}}\rho_{g^{\prime }}\mu_{g^{\prime }g}\nu_{g}P[g(I_{t-1})=g^{\prime }|\Psi (I_{t-1})=1]$.

Next, we consolidate the focal conditional probabilities for every $g\in G_{v}\setminus G_{s}$ at generation *t* into a column vector **p**^(*t*)^. In this vector, **p**^(*t*)^, the *i*_*g*_th entry corresponds to an individual *g* and can be written as ${\left[p^{\left(t\right)}\right]}_{i_{g}}=P[g(I_{t})=g|\Psi (I_{t})=1]$. With this column vector formulation, the master equation (2.4) can now be rewritten in a matrix format. Specifically, we denote by **T** the transition matrix among GRNs $G_{v}\setminus G_{s}$, whose entry at the *i*_*g*_th row and the *i*_*g*′_th column is *ρ*_*g*′_*μ*_*g*′ *g*_*ν*_*g*_ for $g,g^{\prime }\in G_{v}\setminus G_{s}$, i.e. the probability that *g*' transitions into *g* in a single generation or the joint effects of viability selection and mutation. In addition, we can define a second matrix **R** to capture reproductivity, which filters genotypes arising via **T** that have zero reproductive success. More formally, **R** encodes the transition from $g^{\prime }\in G_{v}\setminus G_{s}$ to $g\in G_{v}\cap G_{s}$, whose entry at the *i*_*g*_th row and the *i*_*g*′_th column is again *ρ*_*g*′_*μ*_*g*′ *g*_*ν*_*g*_. Together, **T** and **R** represent the two components of selection in our model, viability and reproduction. With these matrix notations, and including the denominator of equation (2.4) $\nu_{t}\rho_{t-1}=\sum_{g\in G_{v}}\sum_{g^{\prime }\in G}\rho_{g^{\prime }}\mu_{g^{\prime }g}\nu_{g}P[g(I_{t-1})=g^{\prime }|\Psi (I_{t-1})=1]$ where $\sum_{g\in G_{v}}P[g(I_{t})=g|\Psi (I_{t})=1]=1$, the master equation (2.4) becomes

3.1 $$p^{\left(t\right)}=\frac{Tp^{\left(t-1\right)}}{1^{⊤}Tp^{\left(t-1\right)}+1^{⊤}Rp^{\left(t-1\right)}},$$

with $1^{⊤}$ noting a row vector of ones with the proper length. GRN fitness arises from the time-evolution of this master equation.

The matrix **T** plays a key role in the master equation (3.1), and it has a nice property that all its entries are positive. Since **T** corresponds to the transition between GRNs $g,g^{\prime }\in G_{v}\setminus G_{s}$, the relative reproductivity *ρ*_*g*′_ and the viability *ν*_*g*_ are both positive (noting that **R** includes selection via reproduction). Next, we must show that the mutation probability *μ*_*g*′*g*_ is positive as well. Recall that, when constructed through the pathway framework of GRNs, the sub-graph of the genotype network induced by any phenotypic constraint is connected (see §2.3 and electronic supplementary material, appendix C). More formally, the connectedness among GRNs constrained by a non-zero viability and reproductivity implies that, for any $g,g^{\prime }\in G_{v}\setminus G_{s}$, there exists a sequence of mutations which transforms *g*′ to *g* through GRNs in $G_{v}\setminus G_{s}$. Since we assume that any single-locus mutation can occur with a non-zero probability (recall from §2.1), there is a non-zero chance for *g*′ to mutate to *g* within one generation,^2^ i.e. *μ*_*g*′*g*_ > 0. As a result, we observe that **T** is a positive matrix.

For the ease of presentation, we show the convergence of equation (3.1) when the matrix **T** is symmetric and provide the proof for a non-symmetric **T** in electronic supplementary material, appendix D. In this case, the eigenvectors ${\left\{v_{i}\right\}}_{i=1}^{n}$ of the symmetric matrix **T** are linearly independent and form a basis of *n*-dimensional vectors, where $n=|G_{v}\setminus G_{s}|$. We order the eigenvectors such that the magnitudes of their corresponding eigenvalues ${\left\{\lambda_{i}\right\}}_{i=1}^{n}$ are non-increasing. The initial distribution can then be rewritten as a linear combination of the eigenvectors of **T**

3.2 $$p^{\left(0\right)}=\sum_{i=1}^{n}a_{i}v_{i}.$$

In addition, because **p**^(*t*)^ is proportional to **Tp**^(*t*−1)^ for *t* > 0, we have **p**^(*t*−1)^ proportional to **T**^*t*−1^**p**^(0)^ and consequently

3.3 $$\begin{array}{rl} p^{\left(t\right)} & =\frac{T^{t}p^{\left(0\right)}}{1^{⊤}T^{t}p^{\left(0\right)}+1^{⊤}RT^{t-1}p^{\left(0\right)}}=\frac{\sum_{i=1}^{n}a_{i}{\left(\lambda_{i}\right)}^{t}v_{i}}{\sum_{i=1}^{n}a_{i}[{\left(\lambda_{i}\right)}^{t}(1^{⊤}v_{i})+{\left(\lambda_{i}\right)}^{t-1}(1^{⊤}Rv_{i})]}\\ & =\frac{a_{1}v_{1}+\sum_{i=2}^{n}a_{i}{\left(\lambda_{i}/\lambda_{1}\right)}^{t}v_{i}}{a_{1}(1^{⊤}v_{1}+(1^{⊤}Rv_{1}/\lambda_{1}))+\sum_{i=2}^{n}a_{i}[{\left(\lambda_{i}/\lambda_{1}\right)}^{t}(1^{⊤}v_{i})+{\left(\lambda_{i}/\lambda_{1}\right)}^{t-1}((1^{⊤}Rv_{i}/\lambda_{1}))]}, \end{array}$$

where **v**_1_ and *λ*_1_ are the leading eigenvector and the leading eigenvalue of **T**, respectively. Since **T** is a positive matrix, by the Perron–Frobenius theorem, we have |*λ*_1_| > |*λ*_*i*_| for every *i* > 1, which guarantees the convergence of equation (3.1)

3.4 $$\lim_{t\to \infty }p^{\left(t\right)}=\frac{v_{1}}{1^{⊤}v_{1}+(1^{⊤}Rv_{1}/\lambda_{1})}.$$

For a general and potentially non-symmetric matrix **T**, we can first factor **T** by its generalized eigenvectors and its Jordan normal form and then an analogous derivation follows (see electronic supplementary material, appendix D). Therefore, under the pathway framework of GRNs, the master equation (3.1) converges to a stationary distribution that is proportional to the leading eigenvector of **T**. Combined with the GRNs having zero viability/reproductivity, whose probability to be observed under the limit *t* → ∞ can be easily computed given (3.4), the stationary distribution of GRNs describes the balanced scenario between selection, mutation and reproduction.

### 3.2. Case study: binary viability, identical reproductivity and independent mutation

We next turn to a simplified case study to demonstrate the validity of our predicted stationary distribution via derivation and simulation. First, a GRN *g* either always survives the selection stage or becomes inviable, i.e. it has binary viability *ν*_*g*_ ∈ {0, 1}. This assumption also implies that for any GRN $g\in G_{v}$ with a non-zero viability, we have *ν*_*g*_ = 1.

Second, we assume that each GRN g∈G produces the same number of offspring. Equivalently, the probability that an individual randomly sampled from an infinitely large offspring population had a viable parent with a specific GRN *g* is a constant for any viable GRN $g\in G_{v}$. We denote this uniform reproductivity by $\rho_{g}=P[I_{t-1}\to I_{t}|g(I_{t-1})=g,\Psi (I_{t-1})=1]=\rho$, which is non-zero.

Third, given the underlying collection of proteins Ω and genes Γ, the per-generation occurrence of mutation at every γ∈Γ is assumed to be an independent identically distributed Bernoulli random variable with a constant success probability *μ*. Moreover, if it occurs, a mutation at *γ* randomly changes *γ*’s expression behaviour to any other pair of protein activator/product encoded in Ω with an equal probability. Under this assumption of independent and uniform mutation, the per-generation probability that a GRN *g*′ mutates to *g* becomes

3.5 $$\mu_{g^{\prime }g}={\left(\frac{\mu }{|\alpha (\Omega )|-1}\right)}^{d(g^{\prime },g)}{\left(1-\mu \right)}^{\left|\Gamma |-d(g^{\prime },g\right)}\;,$$

where we denote by α(Ω) the set of possible pairs of protein activator/product in Ω, and *d*(*g*′, *g*) is the number of genes with different expression behaviour between *g*′ and *g*.

For this more specific model, we can rewrite the transition matrix **T** into a series

3.6 $$T=T_{0}+T_{1}+T_{2}+\cdots +T_{\left|\Gamma \right|},$$

where the entry at the *i*_*g*_th row and the *i*_*g*′_th column of matrix **T**_*k*_ is *ρμ*_*g*′ *g*_ if *d*(*g*′, *g*) = *k* and 0 otherwise. Observe that **T**_0_ is proportional to the identity matrix **I** (of a proper size), and **T**_1_ is proportional to the adjacency matrix^3^ of the neutral network of GRNs (see §2.3), which we denoted by **A**. Writing ${\tilde{T}}_{k}=T_{k}/\mu^{k}$, whose entries are finite even for a zero per-generation, per-locus mutation probability *μ*, equation (3.6) becomes

3.7 $$\begin{array}{r} T=\rho {\left(1-\mu \right)}^{\left|\Gamma \right|}I+\rho \left(\frac{\mu }{|\alpha (\Omega )|-1}\right){\left(1-\mu \right)}^{|\Gamma |-1}A+\sum_{k=2}^{\left|\Gamma \right|}\mu^{k}{\tilde{T}}_{k}. \end{array}$$

We further consider the scenario that mutations are rare events, specifically, under the limit *μ* → 0. Since the eigenvectors of **I** + *c***A** are exactly the eigenvectors of **A** for any scalar *c*, and ${\left\{T_{k}\right\}}_{k=1}^{\left|\Gamma \right|}$ are symmetric matrices because *d*(*g*′, *g*) = *d*(*g*, *g*′), the theory of eigenvalue perturbation [62,63] ensures that the leading eigenvector of **T** converges to the leading eigenvector^4^ of **A**,

3.8 $$\lim_{\mu \to 0}v_{1}=v_{1}(A).$$

From equation (3.4), we have

3.9 $$\lim_{\mu \to 0}\;\lim_{t\to \infty }p^{\left(t\right)}=\frac{v_{1}(A)}{1^{⊤}v_{1}(A)}.$$

In network science, entries of the leading eigenvector of the adjacency matrix of a connected, undirected graph are known as the eigenvector centrality [64,65] of the nodes. As a result, under the assumptions of binary viability, identical reproductivity and rare uniform mutation, *the probability distribution of viable GRNs converges to a stationary distribution that is proportional to their eigenvector centrality in the neutral network*.

To validate the predicted probability distribution of GRNs under mutation–selection balance, we simulate the evolution of 10^7^ parallel populations. The simulations are parametrized with the constant sets of |Γ|=4 genes and |Ω|=6 proteins. We assume that at least one protein cannot be the product of any gene and whose presence is provided externally. We refer to these externally provided stimuli as *input* proteins. We also assume that at least one protein only has direct physiological effects and cannot activate any of the genes in the network, which we call the *output* proteins. Under this minimal set-up, there are a total of |α(Ω)|=16 potential pairs of expression activator/products (i.e. genes), which leads to |G|=65 536 plausible GRNs. We evolve the populations under the environmental condition such that one of the input proteins is externally provided, and one of the output proteins has a fatal effect (i.e. it must be absent for an individual to have non-zero viability), resulting in $|G_{v}|=45\;389$ possible viable GRNs. Our simulated GRNs sizes were kept small to ensure adequate sampling of the empirical distribution, which grows super-exponentially in the number of genes and proteins. We leave the simulation of larger GRNs to future work.

We simulate the evolution of parallel populations using a Wright–Fisher model [66]. Specifically, we run simulations with 16 individuals in a population,^5^ and given a current generation, the next generation is generated through randomly choosing viable GRNs from the current generation with replacement followed by potential mutations with a per-locus mutation probability *μ* = 0.1. We begin with 10 000 different initial populations where the GRN of every individual is chosen uniformly at random from all possible GRNs G, and 1000 lineages are evolved from each initial population. The 10^7^ parallel populations are evolved for a constant number of generations, and we randomly sample one viable GRN from each of them to form the simulated distribution of GRNs. This fixed length of evolution is determined through the temporal lower bound such that the resulting GRN distribution is theoretically close enough to the stationary distribution within a given level of error tolerance (detailed in electronic supplementary material, appendix E). In electronic supplementary material, figure S7, we show that our approximation holds for population sizes of 1600 and in electronic supplementary material, figure S8 with larger population sizes and much lower mutation rates (*μ* = 0.001).

Moreover, in order to account for the uncertainty of finite-sized sampling in the simulated distribution, we also draw the same number of 10^7^ independent samples from the predicted distribution (3.9) to form an empirical distribution. Repeating the sampling procedure 1000 times, we obtain an ensemble of empirical distributions that captures the effect of finite-sized sampling over the predicted probability that GRNs are to be observed. We further use the averaged variation distance between the empirical distribution and the predicted distribution as the error tolerance from which the number of generations to be simulated is calculated, such that convergence of the model is theoretically guaranteed (electronic supplementary material, appendix E).

**Figure 4.**
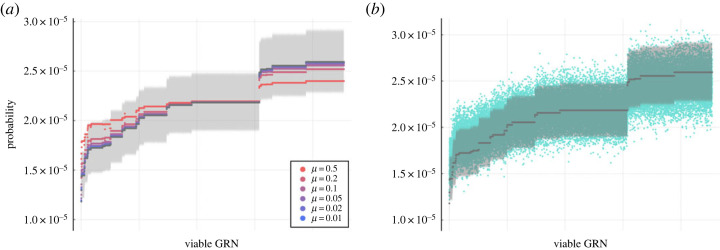
Validation that the evolutionary dynamics of GRNs converges to the derived stationary distribution (3.9). We compare the predicted stationary distribution of viable GRNs under the rare-mutation approximation with (*a*) the exact leading eigenvector of the transition matrix (3.7) with various per-locus mutation probability *μ* (coloured by a red-purple gradient from large to small) and (*b*) the distribution of GRNs sampled from their simulated evolutionary dynamics with *μ* = 0.1 (blue). The predicted distribution under the limit *μ* → 0 is coloured in grey, and the shaded area shows its 95% confidence band that accounts for the uncertainty of finite-sized sampling in the simulations. In both panels, the viable GRNs are ordered increasingly by their predicted probability to be observed.

In figure 4*a*, we compare the exact, properly normalized leading eigenvector of the transition matrix **T** (3.7) along with the predicted stationary distribution of viable GRNs under the rare-mutation approximation (3.9). Observe that even a moderate per-locus mutation probability *μ* leads to a GRN distribution well aligned with the predicted one; especially with respect to the uncertainty arising from finite-sized sampling in the simulations. Moreover, figure 4*b* shows the simulated distribution of viable GRNs after long-term evolution. We see that, despite some overdispersion, the simulated distribution agrees with the derived stationary distribution of GRNs. Direct comparison between the simulated distribution and the exact solution, i.e. the leading eigenvector of the transition matrix (3.7), shows no significant difference as well (see electronic supplementary material, figure S6). Combined, our simulations provide computational evidence that, when viability is assumed binary and mutations are rare, the topology of the neutral network—specifically the eigenvector centrality of mega-nodes—serves as a informative predictor of the distribution of GRNs under mutation–selection balance.

### 3.3. Prevalent gene regulatory networks under mutation–selection balance

We now apply our model to a case study of binary viability, identical reproductivity and rare mutation to further investigate the structure of GRNs that have a higher probability to be observed than others under different selective regimes. We again consider GRNs with a constant collection of six proteins and four genes. In addition, for the ease of presentation, we label the genes by upper-case letter Γ={A,B,C,D} and the proteins by numerals Ω={1,2,3,4,5,6}, where proteins 1 and 2 are the input proteins and proteins 5 and 6 are the output proteins, respectively (see §3.2). Under the pathway framework of GRNs, an environment can be jointly described by: (i) a set of stimuli proteins that are externally provided, (ii) a set of essential proteins whose absence state leads to inviability, and (iii) a set of fatal proteins whose presence state causes inviability. We will focus on seven distinct environments listed in table 1 that showcase the scenarios of single versus multiple stimulated/essential/fatal proteins and their combinations.

**Table 1.** Different environmental conditions specified by the sets of stimulated, essential, fatal proteins.

	Env. 1	Env. 2	Env. 3	Env. 4	Env. 5	Env. 6	Env. 7
stimuli	{1}	{1}	{1, 2}	{1}	{1}	{1, 2}	{1}
essentials	∅	∅	∅	{6}	{5, 6}	{6}	{5}
fatals	{6}	{5, 6}	{6}	∅	∅	∅	{6}

For each of the focal environmental conditions, we examine the prevalent regulatory structures among various groups of GRNs. These groups consist of GRNs satisfying different constraints on their structural properties, which correspond to a few artificially enforced scenarios of interest. Here, the focal topological constraints originate from patterns observed in the most prevalent GRNs, and progressively adding constraints offers a rough ranking of regulatory patterns for their prevalence. We arrange groups of GRNs based on the following four constraints: first, GRNs with a gene of ‘spare’ functionality are excluded, where the spareness of a gene refers to its negligible consequence on the resulting phenotype. This includes self-regulating genes due to the binary state assumption and genes that are activated by an input protein which is not externally stimulated or that produce an output protein without an essential/fatal effect under the given environment. Second, we exclude GRNs with multiple genes of the same, redundant expression behaviour. Third, we only consider those GRNs where all the genes are functionally activated. This constraint mimics the scenario that genes with active expression behaviour are more likely to be observed empirically than inactive ones. Fourth, we exclude GRNs where a gene is directly activated by a stimulus and produces an essential protein to enforce selection on regulation rather than individual genes. Combinations of these four constraints lead to eight distinct groups where the prevalent GRNs are investigated (see table 2).

**Table 2.** Groups of GRNs by imposing constraints on their structural properties.

	no spare genes	no redundant genes	all genes activated	no direct selection
group (i)				
group (ii)	✓			
group (iii)	✓	✓		
group (iv)	✓		✓	
group (v)	✓	✓	✓	
group (vi)		✓		
group (vii)				✓
group (viii)		✓		✓

**Figure 5.**
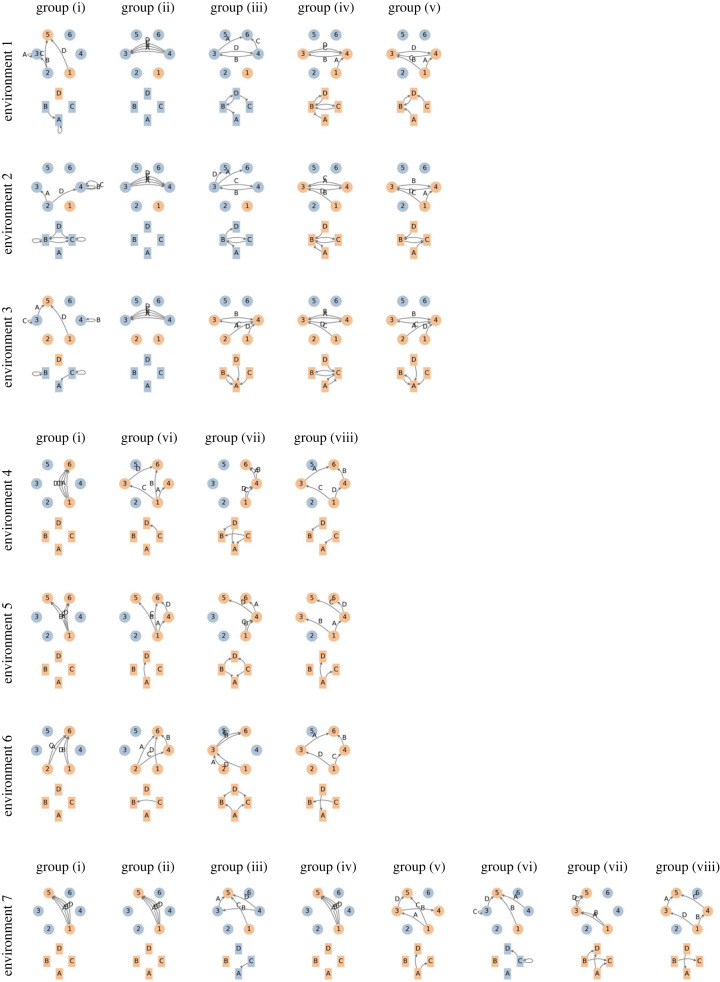
GRN that has the largest eigenvector centrality in the neutral network for different environmental conditions (table 1) and among different constrained groups of GRNs (table 2). For each prevalent GRN, its pathway framework representation is plotted by the circles and the labelled arrows, while its conventional representation is drawn through the rectangles and the unlabelled arrows. A node is coloured in orange if the protein/gene is present/activated and in blue otherwise.

In figure 5, we plot the GRNs that have the largest predicted probability to be observed among the various groups and environments, i.e. the GRNs with the greatest eigenvector centrality in the neutral network under each scenario. Note that such GRNs may not be unique; in fact, one can find multiple similar GRNs through transformations that preserve their roles in the neutral network, e.g. exchanging the expression behaviour of two genes *A* and *B*. Yet, these GRNs all share common structural features, and we only show a random sample from the GRNs with the same, maximal probability to be observed in our prediction. Moreover, figure 5 demonstrates the prevalent GRNs in both the representation of the pathway framework that manifests expression activator/product of each gene (labelled arrows between circles) and that of the conventional notion showing the regulation between genes (unlabelled arrows among rectangles).

A few intriguing observations arise from the prevalent regulatory structure in figure 5. For environmental conditions where protein products can only be fatal (environments 1, 2 and 3), GRNs with many spare genes tend to have the largest predicted probability under mutation–selection balance (group (i)). Once constrained by the absence of the spare genes (group (ii)), we see prevalent GRNs with lots of redundancy where genes share the same expression behaviour or are completely isolated. If we further exclude redundant paths and/or enforce all genes to be activated (groups (iii) and (iv), respectively), the prevalent GRNs demonstrate a structure which seemingly avoids expression activated by the stimulated protein or producing the fatal proteins as much as possible. Interestingly, for the environment with multiple stimuli and constraining on no spare and redundant genes (environment 3 and group (iii)), the functional activeness of all genes naturally emerges.

For the environmental conditions where protein products must be produced for survival (environments 4, 5 and 6), the most prevalent GRNs are the ones where several redundant genes are directly activated by an external stimulus and produce the essential protein (group (i)). When redundant genes are excluded (group (vi)), the prevalent GRNs have multiple parallel paths between the external stimuli and the essential proteins. When constrained such that genes activated by the external stimuli cannot produce the essential protein (group (vii)), the prevalent GRNs similarly show multiple pathways that share the same intermediate genes. Jointly imposing the two constraints mentioned above (group (viii)) results in prevalent GRN structures that maintain multiple, distinct regulatory pathways within the same GRN.

Finally, when both essential and fatal proteins exist (environment 7) and GRNs are unconstrained, the most probable networks only contain redundant genes that are directly activated by the stimulus and synthesize the essential target (groups (i), (ii) and (iv)). When we constrain GRNs such that they cannot have redundant genes, the prevalent regulatory structures typically only have a single pair of connected genes, one that is activated by the external stimuli and whose protein product activates a second gene that produces the essential protein (groups (iii) and (vi)). If we further require the activation of genes or exclude direct selection on individual genes (groups (v), (vii) and (viii)), we again see multiple pathways emerge in the prevalent GRNs.

## 4. Discussion

In this work, we analyse the evolutionary dynamics of GRNs under a quasi-species model with selection, mutation and asexual reproduction. Integrating this model with the pathway framework of GRNs, we analytically show that the population dynamics always converge to a stationary distribution of GRNs given any arbitrary viability function. This stationary distribution characterizes the ensemble of regulatory networks under mutation–selection balance, and it reveals the structural features of GRNs predicted to be favourable under long-term evolution. Next, we investigate a case study assuming binary viability, identical reproductivity and rare mutation, and find that the stationary distribution of GRNs can be derived from the topology of the genotype network alone. Specifically, the probability of observing a GRN under mutation–selection balance is proportional to each GRN’s eigenvector centrality in the neutral network, which—in this simplified model—is a sub-graph of the genotype network consisting of all viable GRNs. Using this approximation, we identify the network structures which are most likely to evolve in response to various selective regimes and regulatory constraints.

Our primary contribution to the broader field of quasi-species theory (and hence population genetics) is a mechanistic explanation for the key assumption of irreducible transition matrices [46–48]. As we mentioned in §1, Moran relates this assumption—which is required for global convergence in quasi-species models—to the scenario that viable genotypes are mutually accessible through mutations [59]. Similarly, a recent review by Aguirre *et al.* [61] concluded that mutational accessibility was present in population genetic models of genotype network evolution where all networks have non-zero fitness. Our work takes an alternative route and assumes that the mechanisms of gene regulation encode the relationship between genotype and phenotype [18]. Despite the simplicity of our model, the mutational accessibility between genotypes with non-zero fitness naturally emerges due to the high dimensionality of GRNs. This result on mutational accessibility implies that quasi-species models will still exhibit global convergence even in cases with extreme fitness values such as occurs in holey adaptive landscapes [67].

Our result that a GRN’s eigenvector centrality in the neutral network is proportional to its fitness sheds light on how we may interpret the prevalence of GRNs under rare mutation and strong selection. When first introduced by Bonacich [64], the eigenvector centrality was designed to capture an individual’s global ‘importance’ as measured by their social ties in a communication network. In particular, the eigenvector centrality is computed based on the idea that a node’s importance is proportional to the sum of its neighbours’ importance scores. This interpretation is nicely translated to the context of the neutral network of GRNs: under mutation–selection balance, the probability of observing a GRN is proportional to the total likelihood of observing its viable, mutational neighbours. As a consequence, the eigenvector centrality contains information on both robustness [68] and evolvability. Additionally, even for simple models, a GRN’s fitness will be a function of both its individual viability/reproductivity and the viability/reproductivity of its neighbours.

The observed prevalent structures of GRNs in our analyses also provide an alternative explanation for evolutionary robustness. We inductively find that prevalent GRNs are those with a minimal number of inviable mutational neighbours (see §3.3). Since the genotype network is a regular graph under the pathway framework of GRNs (recalling from §2.3), i.e. every GRN has the same number of mutational neighbours, GRNs with equal viability and reproductivity can still have different fitness if they have different numbers of *inviable* neighbours. In other words, the observed GRNs under various environmental conditions represent a balance between survival, reproduction and the number of viable neighbours in the neutral network. We emphasize that this concept of robustness emerges naturally from our mechanistic, quasi-species model of GRN evolution rather than an *a prior* assumption about important properties of GRNs.

Previous work has also found that the topological features of genotype networks can be related to evolutionary processes of interest. For example, evolvability has been approximated by the size of the genotype network of a given phenotype [69], as well as the number of ‘neighbouring’ phenotypes inferred from the genotype network [70]. Robustness has been modelled as the node degree in the genotype network [70], and Dall’Olio *et al.* [71] adopted the average path length in the genotype network as a proxy for genetic heterogeneity. To the best of our knowledge, Van Nimwegen *et al.* [68] were the first to bridge between the asymptotic abundance of different genotypes under a population genetic model and their eigenvector centrality in the neutral network. Our study extends Van Nimwegen *et al.*’s [68] conclusion and differentiates a quasi-species perspective from a model lacking genetic variation. For instance, if a population fixes a single genotype at all time and its evolution is modelled as a random walk on the neutral network, results from network science guarantee the fixation probability at a given genotype to be proportional to its *degree* instead of the eigenvector centrality in the neutral network [65].

The current scope of this work is not without limitation. First, we assume a constant, static environment in which the population evolves, whereas populations certainly experience shifting or alternating environmental conditions [72–74]. Consequently, our predicted regulatory structures under mutation–selection balance may not capture all the features present in empirical GRNs [75,76]. Second, our model focused on the joint forces of selection and mutation. Although this model can indeed be extended to include more complicated mechanisms such as recombination [77,78], gene duplication [79,80] and demographics [81], we leave such extensions—along with their possible implications—to future work. Third, when the time scale of environmental change is much faster than that of the evolutionary dynamics (see electronic supplementary material, appendix E), the transient constitution of GRNs in a population shall attract more ‘attention’ than their stationary distribution at mutation–selection balance [82–84]. Put simply, it remains an open question whether real-world populations should ever be conceptualized as at equilibrium (even dynamic) as opposed to existing in some far-from-equilibrium state [85]. Fourth, despite agreement between the derived stationary distribution of GRNs in an infinitely large population and our long-term numerical simulations, we also find that a finite population size moderately influences the transient evolutionary dynamics. Developing a richer understanding of the role genetic drift plays in structuring the evolution of GRNs is an important extension of our work. Finally, despite our model being constrained to the assumptions listed in §2.2, it can be extended to more diverse gene regulatory mechanisms. For instance, gene regulation can be modelled as different logic gates connecting multiple expression activators/suppressors/products, and the chemical states of proteins can also be generalized to continuous dosage. Importantly, the connectivity of the neutral network and its convergence to a stationary distribution still hold under these more complicated models, provided that mutations between GRNs are not prohibited.

Across a broad range of models, our work demonstrates that the neutral network of GRNs is always connected (in agreement with existing computational work [86]) and that the relative frequency at equilibrium of various GRNs can be predicted from first principles. Therefore, our work shows how different evolutionary forces can favour different GRN structures. We believe future work that progressively integrates a broader set of evolutionary mechanisms will result in models capable of being compared with empirical data, and that analytical predictions building from our work will complement existing studies based purely on computational approaches [24,30,36]. Nevertheless, the current work establishes a null expectation for how GRNs are shaped by mutation and selection. Critically, this null expectation appears to recapitulate many of the topological features of molecular interaction networks currently associated with more complex properties like evolvability and robustness. Said differently, our work shows that complex fitness landscapes can emerge from simple evolutionary rules [19].

## Data Availability

The authors affirm that all data necessary for confirming the conclusions of the article are present within the article, figures and tables, and the code of the analyses is available at https://github.com/chiahungyang/GenoNet. The data are provided in the electronic supplementary material [87].

## Endnotes

1 A set *A* is said to be *partitioned* by {*A*_*i*_}_*i*∈*I*_ if $A=\underset{i\in I}{⋃}A_{i}$ and $A_{i}\cap A_{j}=\emptyset$ for two distinct *i*, *j* ∈ *I*.

2 To be more precise, this argument is only valid when the joint probability for any combination of multiple single-locus mutations is non-zero per generation. Otherwise, we can modify the master equation (3.1) by extending the time scale from 1 to Δ*t*, where Δ*t* is the diameter of the sub-graph of the genotype network constrained by a non-zero viability and reproductivity. The modified transition matrix is now proportional to **T**^Δ*t*^, which is a positive matrix since mutation events at different generations are independent. Replacing **T** by **T**^Δ*t*^ we have an analogous derivation to prove the convergence of the master equation.

3 In graph theory, the adjacency matrix **A** is a way to represent the structure of a graph, where *A*_*ij*_ = 1 if nodes *i* and *j* are connected, and *A*_*ij*_ = 0 otherwise.

4 Here we use the notation **v**_1_ (**A**) and *λ*_1_ (**A**) for the leading eigenvector and the leading eigenvalue of **A**, respectively.

5 Given our assumption of no genetic drift, i.e. infinite population size, focusing on the model fit for a small number of individuals is conservative. In addition, we are able to more richly explore the effect of initial conditions and sampling on the resultant distribution of GRNs. This number, i.e. 16, is also comparable to the number of possible expression pairs per locus to facilitate the diversity in the initial populations. In electronic supplementary material, figure S7, we show that our approximation holds for population sizes of 1600 and in electronic supplementary material, figure S8 with larger population sizes and much lower mutation rates.
